# Examining response process validity of script concordance testing: a think-aloud approach

**DOI:** 10.5116/ijme.5eb6.7be2

**Published:** 2020-06-24

**Authors:** Michael Siu Hong Wan, Elina Tor, Judith N. Hudson

**Affiliations:** 1 School of Medicine, The University of Notre Dame Australia, Australia; 2 Faculty of Health and Medical Sciences, University of Adelaide, Australia

**Keywords:** Script concordance testing, response process validity, written think-aloud, assessment, clinical reasoning

## Abstract

**Objectives:**

This study investigated whether medical student responses to Script Concordance Testing (SCT) items represent valid clinical reasoning. Using a think-aloud approach students provided written explanations of the reasoning that underpinned their responses, and these were reviewed for concordance with an expert reference panel.

**Methods:**

A set of 12, 11 and 15 SCT items were administered online to Year 3 (2018), Year 4 (2018) and Year 3 (2019) medical students respectively. Students' free-text descriptions of the reasoning supporting each item response were analysed, and compared with those of the expert panel. Response process validity was quantified as the rate of true positives (percentage of full and partial credit responses derived through correct clinical reasoning); and true negatives (percentage of responses with no credit derived through faulty clinical reasoning).

**Results:**

Two hundred and nine students completed the online tests (response rate = 68.3%). The majority of students who had chosen the response which attracted full or partial credit also provided justifications which were concordant with the experts (true positive rate of 99.6% for full credit; 99.4% for partial credit responses). Most responses that attracted no credit were based on faulty clinical reasoning (true negative of 99.0%).

**Conclusions:**

The findings provide support for the response process validity of SCT scores in the setting of undergraduate medicine. The additional written think-aloud component, to assess clinical reasoning, provided useful information to inform student learning. However, SCT scores should be validated on each testing occasion, and in other contexts.

## Introduction

Clinical reasoning is a cognitive process where the clinician collects information from the history, physical examination and/or investigations related to a patient presentation to come to a conclusion about the patient's health situation. Thereby this process allows for the implementation of appropriate intervention and management.^1^ As diagnostic errors are often related to problems with clinical reasoning, the Accreditation Council for Graduate Medical Education (ACGME) has recently urged educators to include clinical reasoning as a core competency in undergraduate and graduate medical education.^2^ This was in response to relatively slow progress in improving the teaching and assessment of clinical reasoning, including the development of specific assessment tools, as well as research and innovation in clinical reasoning education.^2^ One such explicit tool, Script Concordance Testing (SCT), was described by Charlin in 2000.^3^ It has since been used to assess clinical reasoning in health professional education.^4^^,^^5^ In Medicine, SCT has been implemented in Paediatrics, Neurology, Emergency Medicine and Psychiatry disciplines.^6^^-^^11^ A body of research informing the use of SCT to assess clinical reasoning has gradually developed over the past decade or so.

In each SCT, an authentic but non-complex clinical scenario/vignette is presented and students are asked to assess whether an additional piece of information increases or decreases the probability/appropriateness of the diagnosis, investigation or management in the context of uncertainty.^12^ The additional information could be in the form of further history symptoms, physical examination signs, or investigations or imaging findings.^13^ The response to each item is recorded on a 5-point scale from '-2': much less likely/appropriate; '-1': less likely/appropriate; '0': neither less or more, likely/appropriate; '+1': more likely/appropriate; '+2': much more likely/appropriate.^12^^,^^13^ For example, a 45-year-old man presenting to the Emergency Department with acute onset of chest pain and shortness of breath for 5 hours. The student is asked to determine whether the additional finding of 'unilateral swelling with dilated veins in the right leg' would make the diagnosis of 'pulmonary embolism' much less or less likely, neither less or more likely, more or much more likely.

In order to answer the item, the student will need to activate 'illness scripts' in his or her mind, which have been constructed based on previous clinical encounters/experiences.^14^ In scoring these SCT items, student responses are compared to responses from a panel of experts using the same 5-point scale. The classical aggregated (weighted) scoring system is used.^13^ If a student's response 'concords' with that of the majority of the expert panel (i.e. the modal response of the expert reference panel), a score of '1' (full credit) is awarded reflecting that the consensus reasoning has been applied. A partially weighted credit is awarded if the student's response 'concords' with a minority of the panel, reflecting a difference in interpretation that may still be clinically valuable and worthy of partial credit. Finally, no (0) credit is awarded if none of the experts have chosen the particular response (Table 1).^13^ It is the partially weighted credit in SCT scoring that differentiates it from scoring of classical multiple-choice questions, where only one single best answer response will attract the full one mark. This unique scoring system in SCT acknowledges important real-world clinical situations, where clinicians often interpret data and make alternate clinical decisions, especially under uncertain conditions.

**Table 1:** The formula to calculate the aggregated (weighted) scores for each SCT item

Score Key	-2	-1	0	+1	+2
Number of experts in the panel choosing the response (out of 10)	0	0	1	2	7
Formula	0/7	0/7	1/7	2/7	7/7
Student score	0	0	0.14	0.29	1

While SCT has been shown to be a valid and reliable assessment tool in various examination settings,^15^^-^^18^ recent research has questioned the plausible threats to the validity of the SCT scores, specifically in relation to student and/or expert reference panel member response processing. The classical SCT item format only captures the student's response along the 5-point scale. The actual clinical reasoning and thought processing involved in choosing a particular response is not recorded or examined. To explore student's response processes in the assessment of clinical reasoning, an approach from education, the think-aloud approach, has been applied to health professional education. This approach has proved useful to allow medical, pharmacy and nursing students' thought processes to be examined.^15^^-^^26^ Trainees were asked to either write down or verbalise their thought processes in relation to decision-making when choosing the answer. Pinnock and colleagues found this approach useful in helping both medical students and supervisors learn and teach clinical reasoning in the clinic environment.^24^ In the critical care setting, the think-aloud protocol had been used during ICU rounds to identify strengths and weaknesses concerning the trainees' clinical decision-making processes.^25^ The think-aloud method has also been used to improve the training of community pharmacists when reviewing medications for patient safety.^19^ Johnsen and colleagues noted that the verbal think-aloud approach could help to understand nurses' clinical reasoning in real-life clinical practice and hence provided nurse educators with ways to improve teaching methods in Nursing.^21^ Another study in a paediatric nursing course found that the written think-aloud approach (followed by small group discussions) could foster the learning of clinical reasoning. Students reported increased confidence, as well as valuing the importance of in-depth discussion associated with the items.^27^

Recently, the think-aloud approach has been used to further elucidate the utility of SCT in assessing the clinical reasoning of medical undergraduates and postgraduate trainees.^27^^,^^28^ The process of asking students to justify their reasons for choosing a particular SCT response option, was in response to Kreiter's critique that there is no firm evidence of the clear relationship between the purported construct of the SCT (clinical data interpretation) and the response process of examinees.^29^ Power and colleagues recently used the think-aloud approach to understand the actual response process of paediatric postgraduate trainees in six SCT cases that covered diagnosis, investigation and treatment. They concluded that the written think-aloud approach could identify incorrect clinical reasoning with correct SCT responses, sound clinical reasoning with sub-optimal SCT responses and misinterpretation of the SCT question.^28^ Their study suggested that the think-aloud approach could strengthen the quantitative assessment method provided by the classical SCT. It was valued as an approach providing assessment *for*, as well as *of*, trainees' learning. Indeed, in response to this, Lubarsky and colleagues have suggested think-aloud or concept mapping protocols might also help to shed further light on examinees' use of probability versus typicality-based reasoning strategies in responding to SCT items.^30^

As mentioned above, in SCT, as in all other assessment items in multiple-choice response format, the actual reasoning behind the selection of a particular response option by individual examinees is never clear. The validity of score interpretation is based on the assumption that correct responses by examinees were derived based on appropriate and correct reasoning processes.^31^ Use of the written think-aloud approach offered the potential to explore whether a student's response to each SCT item is underpinned by correct reasoning, consistent with that of the expert reference panel. Thus we applied a similar approach to specifically explore the 'response process' validity of SCT scores in assessing the clinical reasoning of senior medical students.^32^ Unlike Power and colleagues' study,^28^ our study investigated the response process validity of the written think-aloud approach in the under-graduate medical student setting with SCT questions across multiple disciplines.

This study aimed to investigate the 'response process' validity of SCT scores in assessing the clinical reasoning of senior medical students. Students were asked to explain in a text box, the thought process involved in deriving the particular response option selected for each SCT item. The study sought to answer two questions: 1) Are full and partial credit responses from students derived through correct clinical reasoning; and 2) Are responses with no credit indeed a result of faulty clinical reasoning?

## Methods

### Study design

In this descriptive study, a set of 12, 11 and 15 SCT items were administered online to Year 3 (2018), Year 4 (2018) and Year 3 (2019) students, respectively. This was an online test offered by the school's assessment team to prepare students for the year-end summative SCT examination (2018-2019). The SCT items were selected from an item bank of 500 items. The content of each item was mapped to the curriculum of the two clinical years covering Medicine, Surgery, Paediatrics, Psychiatry, Women's Health and General Practice disciplines. Each SCT scenario had been reviewed by the relevant discipline leads and the assessment academics to ensure content validity. The expert panel used for scoring the items consisted of specialists in the relevant disciplines and general practitioners who were directly involved in the teaching of the students. In constructing the final sets of SCT items, any item with inconsistent panelist responses (bi-modal or uniform divergence responses) was modified or discarded to optimise the test before implementing the online test. This step aimed to improve the validity of the assessment tool.^12^

Detailed descriptions of the format of SCT and the scoring system were given to the students at the beginning of the test. To help the student to understand and improve their clinical reasoning and decision-making skills, each student was asked to record online, the reasons behind each of their chosen answers for the SCT items (written think-aloud approach) before choosing the answers according to the 5-point scale.

### Participants

All students in Year 3 and Year 4 from 2018, and in Year 3 in 2019, of the medical program, were invited to participate voluntarily via an announcement on the university's student learning portal. Participant Information was presented online, and consent was obtained by the students clicking the "agree to include the anonymised data for analysis in the study" key. This approach allowed the students to continue to attempt practice SCT items and receive the usual feedback even if their responses were not being collected for this study.

### Data collection

In this study, the online practice tests were delivered via a free online survey tool. The students' answers were compared with those of the expert panel members (n=15). The classical SCT weighted aggregate scoring method was used for scoring.

A full credit was given if a student's response was the same as the expert panel's modal response, and a partial credit was given if a student's response concorded with the minority of the panel according to the formula as shown in Table 1 above. The free text explanation of the clinical reasoning behind choosing each answer was also collected. The keyed responses and explanations data were transferred to a spreadsheet for coding and anonymous analysis. Immediately following the test, students were provided (online) with the responses which attract full and partial credit and the experts' clinical reasoning behind each decision. This was followed by a separate face-to-face feedback session where significantly incorrect clinical reasoning or misinterpretation related to the students' written think-aloud free text entries were explained and discussed with the cohort. This session aimed to improve student clinical reasoning skills and help them to better prepare for the summative examination.

Ethics approval for the study was obtained from the University's Human Research and Ethics Committee (#019023S). All participant data and free text explanations were collected anonymously via the online survey tool.

### Data analysis

Response process validity was quantified as the true positive (TP) rate, i.e. percentage of full and partial credit responses derived through correct clinical reasoning; and true negative (TN) rate, i.e. percentage responses with no credit derived through incorrect/faulty clinical reasoning.

The first author analysed students' free-text justifications for their answers for each of the SCT items. For each SCT item, student's clinical reasoning explanation was compared with the experts' consensus reasoning, to evaluate the extent of concordance between the two, i.e. students and expert clinicians from the reference panel. Students' written think-aloud explanations were coded into six categories: A) Full credit response derived based on correct reasoning, concordant with the experts' reasoning (true positive in full credit responses); B) Partial credit response derived based on correct reasoning, concordant with the experts' reasoning (true positive in partial credit responses); C) Full or partial credit response derived based on incorrect/faulty reasoning as compared with the experts (false positive in both full and partial credit responses); D) Response that received no credit through faulty clinical reasoning (true negative); E) Response that received no credit but free text justification indicates correct reasoning concordant with the experts' reasoning, due to mis-selection of the score keys (false negative); F) Response that received no credit even though free text justification indicates correct reasoning, because none of the expert reference panel members had selected that particular response option (false negative). According to the above categories, the percentage of true positives and true negatives were calculated for the student responses analysed.

## Results

### Students' response process

The participation rate was 68.3% (N = 209). A total of 38 SCT items (12 for Year 3 in 2018; 11 for Year 4 in 2018 and 15 for Year 3 in 2019), with 2,695 student responses were analysed. Of all the 1,679 responses provided by students to each of the SCT items which attracted a full credit (based on the extent of concordance with the expert panel's responses, i.e. the modal response), 1,673 were based on correct clinical reasoning (Category A – True Positives in full credit responses). Of the 700 responses which attracted a partial credit, 696 were based on correct clinical reasoning concordant with the experts (Category B – True Positives in partial credit responses). Ten responses which were awarded full or partial credits were derived based on incorrect/faulty clinical reasoning (Category C – False Positives). Of the 315 responses which attracted no credit, 312 were based on incorrect clinical reasoning (Category D – True Negatives). Two student participants (both in the Year 3 cohorts) had chosen the wrong response option despite correct clinical reasoning due to mis-selection of the wrong answer key (Category E – False Negatives). Three responses had the correct clinical reasoning but received no credit because none of the experts had selected that particular response option (Category F – False Negatives).

As mentioned above, the majority of students who had chosen the answer which attracts full or partial credit also provided justifications which were concordant with the experts (true positive rate of 99.6% for full credit and 99.4% for partial credit answers respectively). The majority of answers that attracted no credit were based on incorrect clinical reasoning (true negative rate of 99.0%).

Examples of students' free-text explanations (direct quotes) of their clinical reasoning with respect to the full or partial credit responses in each Category (A to F) are represented in Appendix 1.

### Other findings

Reviewing the written think-aloud responses as part of the SCT test optimisation process allowed the experts/academics to discuss and modify any items that were flawed or prone to misinterpretation. The following example demonstrates how an SCT item on the investigation was modified after reviewing the written think-aloud explanation by students. The clinical scenario was a 45-year-old man who presents to the Emergency Department with a 3-day history of epigastric pain. The question asked whether the finding of the fact that the pain could be relieved by antacids would make ordering endoscopic examination less or more appropriate. The expert panel's modal answer was 'much less appropriate' (-2) as the procedure is invasive and would only be indicated if the patient had symptoms of anaemia, weight loss or poor response to medical treatment with antacid or proton pump inhibitors. However, the analysis of free-text responses revealed a significant number of students assumed that the patient has recurrence or persistence of epigastric pain; and therefore selected 'more appropriate' (+1) or 'much more appropriate' (+2) where no credits were awarded (Category F). For re-administration of the SCT question, the first presentation of the symptoms was clarified. The clinical scenario was modified to read 'a 45-year-old man presents to the General Practice with a 3-day history of epigastric pain. He has no previous history of similar pain'.

## Discussion

This study sought to explore the response process validity of SCT scores as a proxy measure for the clinical reasoning ability of senior medical students through the written think-aloud approach. Most students seemed to have applied correct clinical reasoning in deriving responses which attract credit in the SCT test. The rate of true positives was 99.6% in full credit responses and 99.4% in partial credit responses. The true negative rate was 99.0%, whereby the students' responses based on faulty clinical reasoning did not earn any credit under the aggregated partial credit scoring model. There is currently no other study in the SCT literature on examinees' response process validity which quantifies the results as the rate of true positives and true negatives.

The fact that a few student responses (6 of 1,679 = 0.4%) had attracted a full credit despite incorrect/faulty clinical reasoning (false positives) suggested there was a potential response process validity threat to SCT scores interpretation due to a construct irrelevant variable. However, the rate of false positives was low. The example in Category C, as presented in the Appendix section, demonstrated that the SCT response format could possibly have a masked misconception by the student, despite the concordance with the expert panel response. Addressing misconceptions such as these in face-to-face discussions, after a written think-aloud approach, can provide students with powerful and timely learning of clinical reasoning. This can also be useful for educators and the expert reference panel to improve questions with ambiguity to avoid confusion and misinterpretation by students.

Recent research highlights that, for more complex and controversial clinical scenarios, the expert panel's modal responses could be variable and even inconsistent over time. Lineberry and colleagues^33^ reported threats to response process validity due to variable expert panel consensus, but this occurred with complex and controversial cases in the postgraduate setting. Variability in the expert panel consensus is less likely with the use of simple classical SCT scenarios/cases.^34^ The latter was used in the current study, and likely explain the very low rate of student discordance with panel responses. Care should also be taken in selecting SCT scenarios, to ensure they introduce sufficient level of 'uncertainty' to fit the conceptual underpinning of SCT (rather than a definitive answer). As described in the Results section, by reviewing the think-aloud responses of the students as part of the SCT test optimisation process, any items that are flawed or confusing can be modified for future administration.

Interestingly, on a few occasions, the students in Year 3 chose the wrong response option despite providing the correct underlying clinical reasoning and interpretation of the item (Category E). This could be due to unfamiliarity with the 5-point response scale of SCT, which could lead to confusion in choosing between the keys of '-2' and '+2' or '-1' and '+1'. More practice in answering SCT items could have minimised this, as this effect was not apparent for the Year 4 test. This is likely due to the fact that Year 4 students had previously been exposed to the SCT format.

Think-aloud is a very useful approach for SCT validation research, particularly in gathering evidence for response process validity in this multiple-choice assessment format. Think-aloud is also a powerful add-on mechanism to improve the educational impact of SCT as one assessment modality in the programmatic assessment. Feedback from the approach can support learners and facilitate further learning. As Power and colleagues demonstrated, in a formative assessment setting, students have the opportunity to better understand the underlying correct clinical reasoning through debriefing/feedback sessions conducted by their teachers.^28^ The rich information potentially provided by SCT can be optimised for learning if care is put into ensuring that the scores reflect what the theory intends. The think-aloud approach and post-scoring debrief offered to students in the current study, provided an example of a counter-measure against validity threats and a stimulus for learning.^34^

A simple short post-test evaluation survey (unpublished) revealed that many participants found that the think-aloud approach with the expert panel's clinical reasoning feedback was helpful for supporting their learning by comparing their answers with the experts. The following anonymous quote from one student illustrated student perception that the debriefing, in explaining the expert panel's reasoning for each SCT item, was useful: by writing the explanation of why the investigation is appropriate and then comparing my thought process with the experts was invaluable for my learning in clinical decision making (Year 4 student).

From a programmatic assessment perspective, if documented systematically and aggregated meaningfully, the rich information from the written think-aloud in SCT can also inform important decision-making for student/trainee progression in training programs.^34^ In high stakes summative examinations using SCT, marking the think-aloud components of the answers (although requiring additional resources for manual marking) may provide additional information in relation to student understanding of a given scenario. In addition to supporting the response process validity of SCT for assessment of medical undergraduates, the approach can facilitate student learning of clinical reasoning.

### Limitations

The study was conducted in one medical school with two years of data (2018-2019) only, and there were limited numbers of SCT items in each test administered. However, score reliability was not as critical in this study, as it aimed to investigate the clinical reasoning underpinning student responses to SCT items, rather than students' overall aggregate scores in each SCT assessment (for pass-fail decisions). The online formative test items were reused from previous years, and therefore, some students may have been exposed to these items if they had been passed on by their senior peers. However, as this was a formative practice opportunity for students and was anonymous, the likelihood of deliberately preparing for such an examination or using an open-book approach was unlikely. In a voluntary setting with a 68.3% participation rate, lower-performing students might be under-represented. However, the very similar average SCT score between the formative test and subsequent summative examination (69% vs 67% respectively) could support the fact that the sampling of the cohort in the current study was representative. The formative nature of this study might limit the interpretation of the results, but to extend such written think-aloud answers in the summative setting without any actual scoring impact on the free text explanation would have been unfair to the students.

This study investigated a phenomenon in its natural setting, i.e. the cognitive process which underpins individual examinees' responses to each SCT item. Data gathered from this study facilitated a better understanding of the written think-aloud approach to answering SCT items in the undergraduate medical program setting, adding to research into clinical reasoning education.

### Future directions

Collaboration with national and international institutions in further research of the think-aloud approach in answering SCT would provide more insight into the response process validity. Further studies using student focus groups could explore students' underlying thought process and thinking, in choosing between the various response-keys on the 5-point scale to ensure the responses are used correctly. There is increasing interest in the use of SCT in medical ethics, and the addition of the think-aloud process to student responses to SCT ethics items would be valuable for later group discussion of ethical dilemmas.^35^

## Conclusions

Although a plausible response process validity threat to SCT score interpretation could arise due to a construct irrelevant variable, this study using a written think-aloud approach in a formative SCT setting in one medical school, demonstrated that the likelihood was relatively low. The finding that the majority of the student keyed-responses corresponded to the correct think-aloud clinical reasoning in various clinical disciplines added further evidence to support the response process validity of SCT scores. The findings have demonstrated that the use of SCT with an additional written think-aloud approach can be a very useful assessment modality for providing rich information to guide further learning.

The study has supported the use of SCT as an explicit tool to assess clinical reasoning in undergraduate medical education. However, validation of SCT scores requires ongoing effort. They should be validated on each testing occasion, and in other contexts.

### Acknowledgements

We would like to acknowledge Miss Eunice Lau for her support in collating the anonymous SCT examination data and Dr Cassy Richmond for her editing input.

### Conflict of Interest

The authors declare that they have no conflict of interest.

## Supplementary material

Supplementary file 1 Appendix 1. Examples of students' free-text explanations (direct quotes) of their clinical reasoning concerning the full or partial credit responses in each Category (A to F)

## References

[1] Krairiksh M, Anthony MK (2001). Benefits and outcomes of staff nurses' participation in decision making.. J Nurs Adm.

[2] Connor DM, Durning SJ, Rencic JJ (2019). Clinical reasoning as a core competency.. Acad Med.

[3] Charlin B, Roy L, Brailovsky C, Goulet F, van der Vleuten C (2000). The Script Concordance test: a tool to assess the reflective clinician.. Teach Learn Med.

[4] Dawson T, Comer L, Kossick MA, Neubrander J (2014). Can script concordance testing be used in nursing education to accurately assess clinical reasoning skills?. J Nurs Educ.

[5] Dumas JP, Blais JG, Charlin B (2015). Script concordance test: can it be used to assess clinical reasoning of physiotherapy student?. Physiotherapy.

[6] Carrière B, Gagnon R, Charlin B, Downing S, Bordage G (2009). Assessing clinical reasoning in pediatric emergency medicine: validity evidence for a Script Concordance Test.. Ann Emerg Med.

[7] Claessens YE, Wannepain S, Gestin S, Magdelein X, Ferretti E, Guilly M, Charlin B, Pelaccia T (2014). How emergency physicians use biomarkers: insights from a qualitative assessment of script concordance tests.. Emerg Med J.

[8] Hamui M, Ferreira JP, Torrents M, Torres F, Ibarra M, Ossorio MF, Urrutia L, Ferrero F (2018). [Script Concordance Test: first nationwide experience in pediatrics].. Arch Argent Pediatr.

[9] Kazour F, Richa S, Zoghbi M, El-Hage W, Haddad FG (2017). Using the Script Concordance Test to evaluate clinical reasoning skills in psychiatry.. Acad Psychiatry.

[10] Lubarsky S, Chalk C, Kazitani D, Gagnon R, Charlin B (2009). The Script Concordance Test: a new tool assessing clinical judgement in neurology.. Can J Neurol Sci.

[11] Talvard M, Olives JP, Mas E (2014). [Assessment of medical students using a script concordance test at the end of their internship in pediatric gastroenterology].. Arch Pediatr.

[12] Wan SH (2015). Using the script concordance test to assess clinical reasoning skills in undergraduate and postgraduate medicine.. Hong Kong Med J.

[13] Lubarsky S, Dory V, Duggan P, Gagnon R, Charlin B (2013). Script concordance testing: from theory to practice: AMEE guide no. 75.. Med Teach.

[14] Charlin B, Brailovsky C, Leduc C, Blouin D (1998). The diagnosis script questionnaire: a new tool to assess a specific dimension of clinical competence.. Adv Health Sci Educ Theory Pract.

[15] Gagnon R, Charlin B, Lambert C, Carrière B, Van der Vleuten C (2009). Script concordance testing: more cases or more questions?. Adv Health Sci Educ Theory Pract.

[16] Humbert AJ, Johnson MT, Miech E, Friedberg F, Grackin JA, Seidman PA (2011). Assessment of clinical reasoning: a script concordance test designed for pre-clinical medical students.. Med Teach.

[17] Nouh T, Boutros M, Gagnon R, Reid S, Leslie K, Pace D, Pitt D, Walker R, Schiller D, MacLean A, Hameed M, Fata P, Charlin B, Meterissian SH (2012). The script concordance test as a measure of clinical reasoning: a national validation study.. Am J Surg.

[18] Wan MS, Tor E, Hudson JN (2018). Improving the validity of script concordance testing by optimising and balancing items.. Med Educ.

[19] Croft H, Gilligan C, Rasiah R, Levett-Jones T, Schneider J (2017). Thinking in pharmacy practice: a study of community pharmacists' clinical reasoning in medication supply using the think-aloud method.. Pharmacy (Basel).

[20] Forsberg E, Ziegert K, Hult H, Fors U (2014). Clinical reasoning in nursing, a think-aloud study using virtual patients - a base for an innovative assessment.. Nurse Educ Today.

[21] Johnsen HM, Slettebø Å, Fossum M (2016). Registered nurses' clinical reasoning in home healthcare clinical practice: a think-aloud study with protocol analysis.. Nurse Educ Today.

[22] Lee J, Lee YJ, Bae J, Seo M (2016). Registered nurses' clinical reasoning skills and reasoning process: a think-aloud study.. Nurse Educ Today.

[23] McAllister M, Billett S, Moyle W, Zimmer-Gembeck M (2009). Use of a think-aloud procedure to explore the relationship between clinical reasoning and solution-focused training in self-harm for emergency nurses.. J Psychiatr Ment Health Nurs.

[24] Pinnock R, Fisher TL, Astley J (2016). Think aloud to learn and assess clinical reasoning.. Med Educ.

[25] Siddiqui S (2014). 'Think-aloud' protocol for ICU rounds: an assessment of information assimilation and rational thinking among trainees.. Med Educ Online.

[26] Verkuyl M, Hughes M, Fyfe MC (2018). Using think aloud in health assessment: a mixed-methods study.. J Nurs Educ.

[27] Tedesco-Schneck M (2019). Use of script concordance activity with the think-aloud approach to foster clinical reasoning in nursing students.. Nurse Educ.

[28] Power A, Lemay JF, Cooke S (2017). Justify your answer: the role of written think aloud in script concordance testing.. Teach Learn Med.

[29] Kreiter CD (2012). Commentary: the response process validity of a script concordance test item.. Adv Health Sci Educ Theory Pract.

[30] Lubarsky S, Gagnon R, Charlin B (2012). Script concordance test item response process: the argument for probability versus typicality.. Adv in Health Sci Educ.

[31] Kane MT (2013). Validating the interpretations and uses of test scores.. Journal of Educational Measurement.

[32] Cook DA, Beckman TJ (2006). Current concepts in validity and reliability for psychometric instruments: theory and application.. Am J Med.

[33] Lineberry M, Hornos E, Pleguezuelos E, Mella J, Brailovsky C, Bordage G (2019). Experts' responses in script concordance tests: a response process validity investigation.. Med Educ.

[34] Wan SH, Tor E, Hudson JN (2019). Commentary: expert responses in script concordance tests: a response process validity investigation.. Med Educ.

[35] Pau A, Thangarajoo S, Samuel VP, Wong LC, Wong PF, Matizha P, et al. Development and validation of a script concordance test (SCT) to evaluate ethical reasoning ability among first and fifth year students in a medical school. J Acad Ethics. 2019;17(2):193-204.

