# Supplementary material for: Examining response process validity of script concordance testing: a think-aloud approach
Source: Int J Med Educ. 2020 Jun 24;11:127–35. doi: 10.5116/ijme.5eb6.7be2 (PMC7870454; doi:10.5116/ijme.5eb6.7be2)
Supplement: Supplementary file 1 — Appendix 1. Examples of students' free-text explanations (direct quotes) of their clinical reasoning concerning the full or partial credit responses in each Category (A to F) [file ijme-11-127-S1.pdf]

Appendix 1.

Examples of students' free-text explanations (direct quotes) of their clinical reasoning concerning the full or partial credit responses in each Category (A to F)

1.1. Example of Category A/B responses: a full or partial credit response from students with clinical reasoning in concordance with the panel.

Clinical scenario

A 25-year-old man presents to the Emergency Department with chest pain and shortness of breath. On examination, his BP is 110/90 mmHg, pulse 120/min.

If you were thinking of the diagnosis:	And then you find:	The diagnosis is:	
Pulmonary embolism	His left leg is swollen with dilated veins.	A B C D E<br>-2 -1 0 +1 +2	-2 = much less likely<br>-1 = less likely<br>0 = neither less nor more likely<br>+1 = more likely<br>+2 = much more likely

The majority of the experts in the panel had chosen a 'much more likely' response as the presence of a unilateral swollen leg with dilated veins was highly suggestive of a deep venous thrombosis (DVT) and therefore could proceed to pulmonary embolism (PE). One student's free-text response included swollen left leg with dilated veins is suggestive of DVT. A DVT predisposes to PE, which was concordant with the experts.

✠ ✠ ✠ ✠ ✠ ✠ ✠ ✠ ✠ ✠ ✠ ✠ ✠ ✠ ✠

1.2. Example of Category C response: clinical reasoning not in concordance with the expert panel despite a full credit response from the student.

Clinical scenario

A 45-year-old presents to the Emergency Department with a 3-day history of epigastric pain.

If you were thinking of ordering:	And then you find:	The investigation is:	
ECG	The pain radiates to his left shoulder	A B C D E<br>-2 -1 0 +1 +2	-2 = much less appropriate<br>-1 = less appropriate<br>0 = neither less nor more appropriate<br>+1 = more appropriate<br>+2 = much more appropriate

`Expert panel's most frequently selected (modal) response was 'much more appropriate' as the patient would likely be having cardiac angina type of pain and therefore an ECG would be indicated to rule in or rule out cardiac ischaemia.

However, a Year 4 student thought that 'the pain radiating to the left shoulder is suggestive of diaphragmatic involvement, which could be due to pericarditis'; and therefore chose 'much more appropriate'; which was an incorrect clinical concept as pericarditis does not typically result in shoulder pain nor involve the diaphragm anatomically.

✠ ✠ ✠ ✠ ✠ ✠ ✠ ✠ ✠ ✠ ✠ ✠ ✠ ✠ ✠

1.3. Example of Category D response: Student response that received no credit with clinical reasoning not concordant with an expert panel

Clinical scenario

A 64-year-old man presents with an episode of jaundice. He has denied any discomfort but is feeling itchy and lethargic.

If you were thinking of the following action:	And then you find:	The investigation is:	
Ordering a CT Abdomen	EBV IgM is elevated	A B C D E<br>-2 -1 0 +1 +2	-2 = much less appropriate<br>-1 = less appropriate<br>0 = neither less nor more appropriate<br>+1 = more appropriate<br>+2 = much more appropriate

The expert panel's most frequently selected (modal) response was 'much less appropriate' as the patient's jaundice was most likely due to acute EBV infection resulting in raised liver enzymes. CT abdomen was not useful in this presentation and would expose the patient to unnecessary radiations. However, a Year 4 student explained that 'EBV was often associated with gastric carcinoma and therefore a CT abdomen would be very appropriate to confirm the carcinoma in the stomach'; for which the clinical concept was incorrect.

✠ ✠ ✠ ✠ ✠ ✠ ✠ ✠ ✠ ✠ ✠ ✠ ✠ ✠ ✠

1.4. Example of Category E response: clinical reasoning from student in concordance with the majority of the panel but the wrong response option key selected.

Clinical scenario

A 32-year-old woman presents with a 2-day history of mild cramping lower abdominal pain and light vaginal bleeding. Her last normal menstrual period was 6 weeks ago.

If you were thinking of the diagnosis:	And then you find:	The diagnosis is:	
Ectopic pregnancy	her serum beta HCG is 3000 IU and there is no intrauterine pregnancy identified on trans-vaginal scan	A B C D E<br>-2 -1 0 +1 +2	-2 = much less likely<br>-1 = less likely<br>0 = neither less nor more likely<br>+1 = more likely<br>+2 = much more likely

The expert panel's consensus reasoning behind the response of 'much more likely' was that a raised serum beta HCG indicated pregnancy and the transvaginal ultrasound scan (TVS) findings of the absence of intrauterine pregnancy, made the diagnosis of an ectopic pregnancy much more likely. However, a Year 3 student chose the response option of "-2" (much less likely) with a free text entry of 'beta HCG positive makes pregnancy likely, and none identified on TVS makes ectopic much more likely'; which was the consensus clinical reasoning. The student had most likely clicked the wrong key response inadvertently.

✠ ✠ ✠ ✠ ✠ ✠ ✠ ✠ ✠ ✠ ✠ ✠ ✠ ✠ ✠

1.5. Example of a Category F response: Clinical reasoning from student in concordance with the majority of the panel but response received no credit because none of the experts had selected that particular answer option.

Clinical scenario

A 45-year-old man presents to the Emergency Department with a 3-day history of epigastric pain.

If you were thinking of the following action:	And then you find:	The investigation is:	
Ordering a Chest X-ray	bronchial breath-ing and crackles on right lower chest	A B C D E<br>-2 -1 0 +1 +2	-2 = much less appropriate<br>-1 = less appropriate<br>0 = neither less nor more appropriate<br>+1 = more appropriate<br>+2 = much more appropriate

The expert panel's unanimous response was 'much more appropriate (+2)' as the clinical signs were typical of lobar pneumonia and therefore, a chest X-ray would be much more appropriate in this clinical setting. As a result, no expert in the panel chose 'more appropriate (+1)', and this answer key, as well as the rest (0, -1, -2), did not attract any mark in the item using the classical aggregated scoring method. However, a few student participants had the appropriate clinical reasoning explanation in the free text, i.e. diagnosing lobar pneumonia, and chose 'more appropriate (+1)' to order the chest X-ray as the investigation, hence scoring a zero score for the question.
